# Diphtheria-Associated Myocarditis: Clinical Profiles and Mortality Trends in a Tertiary Care Hospital in Pakistan

**DOI:** 10.7759/cureus.56744

**Published:** 2024-03-22

**Authors:** Saadia Ilyas, Imran Khan, Zaland A Yousafzai, Qazi Kamran Amin, Zainab Rahman, Muhammad Bilal

**Affiliations:** 1 Pediatric Cardiology, Lady Reading Hospital, Peshawar, PAK; 2 Cardiology, Lady Reading Hospital, Peshawar, PAK; 3 Medicine, Lady Reading Hospital, Peshawar, PAK; 4 Pediatric Medicine, Lady Reading Hospital, Peshawar, PAK

**Keywords:** diphtheria, infectious disease, cardiology, pediatric infectious disease, cardiac complications

## Abstract

Background: *Corynebacterium diphtheriae* infection, causing diphtheria, is a public health concern, particularly in developing nations like Pakistan. Despite immunization efforts, recent outbreaks since 2022 have emphasized the continuing threat. This study focuses on describing the clinical characteristics of children with diphtheria-induced myocarditis and exploring the association between early cardiac abnormalities, future fatality rates, and contributing factors.

Methods: A one-year cross-sectional study was undertaken at Lady Reading Hospital MTI Peshawar, encompassing 73 pediatric patients diagnosed with diphtheria-associated myocarditis. Data, including demographic characteristics, cardiac enzymes, and serial ECG and echocardiography data, were gathered from the health management information system (HMIS). Institutional Ethical Committee approval was obtained, and informed consent was waived due to its retrospective nature.

Results: Gender distribution within the study was balanced, with 35 males (47.9%) and 38 females (52.1%). ECG data revealed various prevalence rates: 27.4% for rhythm abnormalities, 20% for conduction abnormalities, 6.8% for ischemia alterations, and 20.5% for normal findings. Treatment measures included anti-diphtheria serum (ADS) in 87.7% and temporary pacemaker placement (TPM) in 13.7% of patients. Echo findings indicated a variety of cardiac dysfunctions: 53.4% with no dysfunction, 9.6% mild malfunction, 6.8% with moderate dysfunction, and 30.1% with severe dysfunction. The categorization of creatine kinase (CK), lactate dehydrogenase (LDH), and troponin I (Trop I) gave insights into the biochemical aspects.

Conclusion: This study gives a full insight into the clinical symptoms of diphtheria-induced myocarditis in children. The findings can help establish a foundation for ongoing study into potential gender-related trends in clinical outcomes, contributing to improved care and preventative methods.

## Introduction

*Corynebacterium diphtheriae* infection, caused by the bacterium of the same name, results in diphtheria, a potentially severe respiratory illness preventable through vaccination. Despite a decline in its overall incidence, diphtheria remains endemic in numerous developing nations, like Pakistan, with a recent outbreak in 2022. Toxigenic strains of *C. diphtheriae* instigate this ailment, an infectious disease with the potential for a fatality that can be effectively prevented through vaccination [1]. In Pakistan, according to the World Health Organization, the annual number of cases of diphtheria has increased since 2015 [2]. Despite the initiation of diphtheria toxoid in routine childhood vaccinations as part of the Extended Program of Immunization in 1978, administered to children at 6, 10, and 14 weeks of age, the Ministry of Health Services, Regulations, and Coordination (NHS, R&C) reported that in the first three quarters of 2022, there were 342 recorded cases of diphtheria causing 39 fatalities, mostly in the pediatric population [3]. According to the K-P Health Department, there are presently 259 suspected cases across the province, and it is estimated that as much as 80% of the patients were pediatric cases [4]. Children under the age of 15 are most commonly affected by diphtheria, and multiple studies have indicated that populations with compromised immunity and individuals who are unimmunized are more susceptible to the disease [5].

Cardiac complications, specifically myocarditis, occur in approximately 10-25% of patients with respiratory diphtheria [6]. The most frequent cause of death from diphtheria is cardiac involvement, which has a 50-75% mortality rate [7].

Cardiac manifestations can be variable and include myocardial dysfunction, rhythm abnormalities (bradyarrhythmias or tachyarrhythmias), and heart blocks requiring pacing. Elevated cardiac enzymes and ECG abnormalities may indicate asymptomatic myocardial involvement [8].

The present study was aimed at identifying the clinical profiles of patients with diphtheria-induced myocarditis and evaluating the correlation between initial cardiac findings, subsequent mortality rates, and the factors contributing to adverse outcomes at a tertiary care hospital in Peshawar.

## Materials and methods

Study design

A longitudinal cross-sectional investigation was undertaken at Lady Reading Hospital MTI Peshawar, encompassing pediatric patients who had been diagnosed with diphtheria-associated myocarditis, for a duration of one year from January 2023 to January 2024.

Setting

The pediatric cardiology department at Lady Reading Hospital (MTI) Peshawar, which is a 1797-bed main tertiary healthcare facility in Khyber-Pakhtunkhwa province of Pakistan. Pediatric cardiology is one of the 33 state-of-the-art departments of the hospital receiving 200 patients in the outpatient department and referrals on a daily basis, the department is equipped with dedicated facilities for electrocardiogram (ECG), echocardiogram, cardiac magnetic resonance imaging (MRI), and computed tomography (CT), stress testing and Holter monitoring facilities.

Participants

All diphtheria-positive pediatric patients with ages ranging from one year to 17 years who developed cardiac complications and were referred to the pediatric cardiology department were included in the study. Pediatric patients who had no cardiac complications secondary to the diphtheria infection were excluded.

Study size

A total of 73 patients were included in the study retrospectively, admitted over the course of one year from December 2022 to December 2023.

Data collection

The patient data was collected using the Health Management Information System (HMIS). Patients’ demographic data, along with cardiac enzymes and data including serial ECGs and echocardiogram data, were collected and recorded in a Microsoft Excel spreadsheet (Microsoft® Corp., Redmond, WA).

Variables and cardiac assessment

Besides the demographic data of the patients like age and gender, the following variables during the cardiac assessment of the patients were recorded:

ECG Results

Twelve-lead ECGs were taken when the patient was admitted and continuously followed during their hospitalization. Alterations in rhythm, conduction irregularities, and deviations in ST segments were recorded. Instances of complete heart block (CHB), sinus node disease with junctional escape rhythm, bundle branch blocks (RBBB, LBBB), and first-degree block were classified as conduction system abnormalities in the ECGs, which were recorded and classified into four categories based on the findings. The second category captured rhythm abnormalities such as ventricular tachycardia (VT), sinus arrhythmia, tachycardia, and bradycardia. The third group focused on ischemia alterations, notably highlighting ST elevation and ST depression. The last category included normal results and also encompassed cases with rhythm irregularities that lacked clear categorization.

Echocardiographic Evaluation

Echocardiograms were performed at admission and repeated at discharge. Left ventricular ejection fraction (LVEF) and the grade of dysfunction were assessed to quantify cardiac involvement. Echocardiogram dysfunction was categorized as mild dysfunction, defined as LVEF ranging from 40% to 49%. Moderate dysfunction corresponds to an LVEF ranging from 30% to 39% and severe dysfunction is defined as an LVEF of less than 30%.

Cardiac Enzymes

Tests of cardiac biomarkers, including troponin-I (Trop-I), creatine kinase-MB (CK-MB), and lactate dehydrogenase (LDH), were undertaken to identify the amount of myocardial injury.

Statistical methods

The collected data was cleaned of all errors prior to data analysis. The data was analyzed using the Statistical Package for the Social Sciences (IBM SPSS Statistics for Windows, IBM Corp., Version 22.0, Armonk, NY). Descriptive statistics were applied to demographic data and categorical variables like gender, age, and age group. Other continuous and categorical variables were distributed among the gender groups and the percentage was calculated. Inferential analysis was used to measure the association between the quantitative variables in the data, i.e., mortality, ejection fraction on admission and discharge, and cardiac enzymes like Trop-I and CK-MB levels.

## Results

A total of 73 patients were included in the study. Around 47.9% (35) were males and 52.1% (38) were females. The mean age was nine with a standard deviation (SD) of 3, and gender distribution was uniform among the participants, males averaged 10 years old with an SD of 3, while participants who were female averaged nine years old with an SD of 3. The mean ejection fraction (EF%) was 52 with an SD of 17. The mean CK level is 398 (SD 320). LDH values, on the other hand, exhibited a mean of 469 with an SD of 208, while the mean level of Trop-I was 5, with an SD of 9 (Table 1).

**Table 1:** Gender-based distribution of quantitative variables EF: ejection fraction, CK: creatine kinase, LDH: lactate dehydrogenase, Trop I: troponin I

Variables		Male		Female		Total	
	Reference values and units	Mean	Standard Deviation	Mean	Standard Deviation	Mean	Standard Deviation
Age	in Years	10	3	9	3	9	3
EF%	52-74%	52	17	52	18	52	17
CK	≤90 U/l	372	314	422	328	398	320
LDH	14-280 U/l	486	209	455	210	469	208
Trop I	<1 ng/l	5	9	5	9	5	9

Table 2 provides important information on the patient population's demographics, therapy approaches, and clinical presentations.

**Table 2:** Gender-based distribution of categorical variables ADS: anti-diphtherial serum, TPM: temporary pacemaker, LDH: lactate dehydrogenase, CK: creatinine kinase, Trop I: Troponin I, ECG: electrocardiogram, ECHO: echocardiogram

Variables		Male		Female		Total	
		Count	Percent	Count	Percent	Count	Percent
Gender	Male	35	47.9%	0	0.0%	35	47.9%
	Female	0	0.0%	38	52.1%	38	52.1%
	Total	35	47.9%	38	52.1%	73	100.0%
ECG Findings	Conduction Abnormalities	7	9.6%	13	17.8%	20	27.4%
	Rhythm Abnormalities	20	27.4%	13	17.8%	33	45.2%
	Ischemic Changes	5	6.8%	0	0.0%	5	6.8%
	Normal Findings	3	4.1%	12	16.4%	15	20.5%
ADS Received	ADS Received	35	47.9%	29	39.7%	64	87.7%
	ADS Not Received	0	0.0%	9	12.3%	9	12.3%
TPM	TPM Needed	4	5.5%	6	8.2%	10	13.7%
	TPM Not Needed	31	42.5%	32	43.8%	63	86.3%
Dischage ECHO	Normal	18	24.7%	20	27.4%	38	52.1%
	Mild Dysfunction	0	0.0%	0	0.0%	0	0.0%
	Patient Expired	17	23.3%	15	20.5%	32	43.8%
	Moderate Dysfunction	0	0.0%	3	4.1%	3	4.1%
Outcome	Discharged	19	26.0%	23	31.5%	42	57.5%
	Expired	16	21.9%	15	20.5%	31	42.5%
ECHO LV Dysfunction	No Dysfunction	20	27.4%	19	26.0%	39	53.4%
	Mild Dysfunction	2	2.7%	5	6.8%	7	9.6%
	Moderate Dysfunction	4	5.5%	1	1.4%	5	6.8%
	Severe Dysfunction	9	12.3%	13	17.8%	22	30.1%
CK	Normal	10	13.7%	8	11.0%	18	24.7%
	Raised	25	34.2%	30	41.1%	55	75.3%
LDH	Normal	19	26.0%	21	28.8%	40	54.8%
	Raised	16	21.9%	17	23.3%	33	45.2%
Trop I	Normal	16	21.9%	22	30.1%	38	52.1%
	Raised	19	26.0%	16	21.9%	35	47.9%

There was a balanced gender distribution among the 73 participants, with 35 (47.9%) men and 38 (52.1%) women. A wide spectrum of cardiovascular issues was shown by the ECG results: 20.5% showed normal findings, 45.2% indicated rhythm abnormalities, 6.8% exhibited ischemia alterations, and 27.4% showed conduction abnormalities. Thirteen percent of patients required a temporary pacemaker (TPM), and 87% of patients received anti-diphtherial serum (ADS). Echocardiogram results obtained at the time of patient discharge revealed that, despite 43.8% of patients having expired and no echo performed, 52.1% of patients had a normal echo, which is nearly identical to the normal echo reported at the time of admission. This indicates that nearly half of the patients with abnormal echo contribute to mortality. Furthermore, the analysis of cardiac enzymes revealed that, with the exception of CK, 24.7% of the patients had normal levels of CK, 54.8% of the samples had normal levels of LDH, and 52.1% of the samples had normal levels of Trop-I. These results of the cardiac enzymes nearly balanced out the abnormal values.

**Figure 1:**
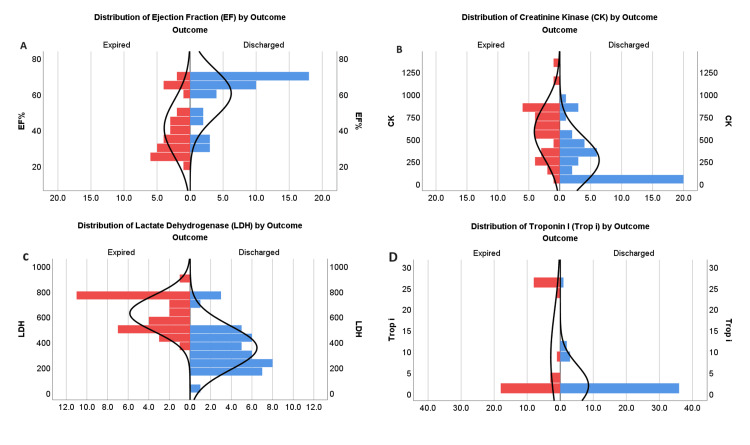
Graphical representation of inferential analysis of the association among variables: Panel A shows the association of EF with the outcome, panel B CK with the outcome, panel C LDH with the outcome, and panel D shows trop I with the outcome. EF: ejection fraction, CK: creatine kinase, LDH: lactate dehydrogenase, Trop I: troponin I

The findings displayed in Table 3 clarify the strong correlations that exist between cardiac enzymes, treatment modalities, and clinical outcomes. The results for the patients who were given a TPM showed a sharp difference; 10 of them required TPM, but none of them expired. This resulted in a statistically significant P value of 0.000. In contrast, among the 63 patients who did not require a TPM, there was a significant variation, with 42 normal discharges and 21 deaths. Correlating the biochemical indicators, such as LDH, CK, and Trop-I, demonstrates strong correlations with clinical outcomes. As for CK levels, the P value of 0.000 showed that the 18 individuals with normal CK levels had a considerably higher chance of being discharged. On the other hand, the mortality rate was significantly higher among the 55 individuals who had elevated levels of CK. A similar pattern was observed for LDH levels, with a P value of 0.000 indicating that the 40 patients with normal LDH levels had a considerably greater normal discharge rate than the 33 patients with elevated LDH levels who expired. Considering Trop-I levels, the relationship with outcome is also significant. As indicated by the P value of 0.000, patients with normal Trop-I levels (a total of 38) showed a considerably better likelihood of being normally discharged. On the other hand, there was an apparent increase in mortality among the 35 patients who had elevated Trop-I levels (Figure 1).

**Table 3:** Inferential analysis of outcome with other variables showing significant association using Chi-square test TPM: temporary pacemaker, CK: creatine kinase, LDH: lactate dehydrogenase, Trop I: troponin I P-value less than 0.05 was considered as significant.

Variables		Outcome		Total	P value
		Discharged	Expired		
TPM	TPM Needed	0	10	10	0.000
	TPM Not Needed	42	21	63	
CK	Normal	17	1	18	0.000
	Raised	25	30	55	
LDH	Normal	35	5	40	0.000
	Raised	7	26	33	
Trop I	Normal	33	5	38	0.000
	Raised	9	26	35	

## Discussion

About 10% to 25% of individuals who have respiratory diphtheria will develop cardiac complications, notably myocarditis [6]. The most common cause of death from diphtheria is cardiac involvement, which has a mortality rate that ranges from 50% to 75% [7].

Diphtheria toxin (DT) induced breakdown of actin filaments is key as the toxin is known to produce severe, often fatal, cardiomyopathy linked with diphtheria, 60-70% of mortality during the acute phase of diphtheria has been linked to myocarditis [9]. Tachyarrhythmias, bradyarrhythmia, and total heart block are all brought on by DT in addition to myocardial dysfunction. Acute inflammation of the sinoatrial and atrioventricular nodes can cause conduction system abnormalities, which are indicators of serious myocardial injury and can be deadly even with ventricular pacing [10].

Extension of the PR interval, alterations in the ST-segment, disappearance of anterior R waves, bundle branch block, total heart block, and arrhythmias of the supraventricular and ventricular regions are common among the ECG findings. Elevated cardiac enzymes and ECG alterations can identify asymptomatic myocardial involvement [11]. According to the findings of our research, the most prevalent cardiac ECG findings were rhythm abnormalities. These rhythm abnormalities included sinus arrhythmias, bradycardia, and tachycardia present in roughly 45.2% of the patients. The prevalence of bradyarrhythmias was higher in our research. The second most frequent ECG finding consisted of conduction abnormalities. Significant conduction abnormalities, such as total heart block and bundle branch block, were linked to a poor prognosis, as has been previously demonstrated by other studies [12].

Our research findings revealed that among the 10 patients with CHB needing pacemaker placement, all 10 patients experienced a fatality. This correlates with the findings of Keen et al., who revealed a substantial link between pacemaker reliance and mortality [12]. Additionally, this trend is consistent with the results of research by Stockton et al., where all patients requiring pacing ultimately succumbed to their disease [13], this can be attributed to the late presentation of patients in our setup.

In addition, a significant increase in the levels of cardiac enzymes is linked to the development of severe heart failure. CPK-MB and cardiac troponin levels, which have a substantial correlation with cardiac mortality, may be helpful outcome predictors. In a study conducted in 2018, cardiac Trop-I had the best sensitivity (80%) while CK-MB had the highest specificity (95.56%) [14]. Unfortunately, the link between Trop-I and death rates is still not well understood [15]. The Trop-I level is likely a reflection of the severity of the disease as well as the damage that has been done to the myocardium; this is supported by our results in which mortality was significantly higher in patients with raised cardiac biomarkers LDH, CK, and Trop-I.

Echocardiography is a useful non-invasive method for assessing heart function. However, due in part to limited access to resources and knowledge, its use in the diagnosis and treatment of diphtheria has been restricted, especially in low- and middle-income countries (LMICs) [16-18]. There is a substantial knowledge vacuum about echocardiographic findings in cases of diphtheria myocarditis. Despite the absence of overt clinical indications of heart failure, people with severe diphtheria may display insignificant abnormalities in cardiac function and subclinical dysrhythmias. Abnormalities in echocardiography include diastolic and systolic dysfunction, which is defined by a significant decrease in LVEF [19]. Furthermore, pericardial effusion, mitral and tricuspid valve regurgitation, left ventricular dilatation, and increased LV wall thickness can all be seen on echocardiograms [17,19]. An array of cardiac dysfunctions was identified in the study population based on the echocardiographic evaluation of LV function. A significant proportion of the cohort, specifically 53.4%, of the patients demonstrated no dysfunction. Mild dysfunction was observed in 9.6% of cases, while moderate dysfunction was evident in 6.8%. Notably, severe dysfunction, demonstrating a significant impairment in LV contractility, was identified in 30.1% of the study participants. The results highlight the various heart-related symptoms linked to myocarditis caused by diphtheria. The presence of different levels of dysfunction indicates a significant influence on the heart physiology of those afflicted. Understanding the pattern of LV dysfunction is essential for customizing suitable treatments and predicting outcomes in the treatment of diphtheria-related heart issues. The results emphasize the importance of closely monitoring and implementing specific therapies, especially in situations of severe LV failure, to reduce negative outcomes and enhance patient prognosis.

The histopathologic characteristics of myocarditis caused by diphtheritis have only been examined in autopsy series [20]. The cardiac autopsy specimen revealed active inflammation in the interstitial spaces and large regions of myocardial necrosis and hyaline degeneration. Infiltrates of mononuclear cells containing eosinophilic cytoplasm are also discovered in these regions. Tissue sections stained with fluorescent antibodies show that the DT is distributed unevenly throughout the cardiac fibers, which may help to explain how the toxin contributes to myocardial injury. Electron micrography demonstrates significant ultrastructural changes within implicated myofibers largely involving the mitochondria which appear enlarged with loss of matrix and disordered cristae which is also connected with the depletion of glycogen and accumulation of lipid droplets [20].

The current approach to treating diphtheria myocarditis mostly focuses on supportive care with the goal of preserving normal hemodynamic parameters. Antiarrhythmic medications are generally saved for cases of persistent tachyarrhythmias. While early therapy for some arrhythmias that are at high risk of development or are linked to unfavorable outcomes is required, prophylactic treatment of subclinical arrhythmias is not advised [21]. Patients suffering from bradyarrhythmia and severe diphtheria myocarditis may benefit from TPM implantation.

Limitations

Limitations include probable incomplete data collecting from medical records and biases inherent in the retrospective methodology. Broader population generalizability may be limited by the study's single-center design.

## Conclusions

This thorough investigation highlights the intricacy of the illness and the significance of close cardiac surveillance by offering vital insights into the cardiac symptoms of myocarditis caused by diphtheria. The degree of cardiac involvement is demonstrated by the pacemaker placement and in-hospital mortality associations that have been observed. Additional research is necessary to validate these findings and examine strategies that may improve outcomes in high-risk populations.
